# Patient predictors of poor drug sensitive tuberculosis treatment outcomes in Kyiv Oblast, Ukraine

**DOI:** 10.12688/f1000research.12687.3

**Published:** 2019-11-22

**Authors:** Omowunmi Aibana, Andrej Slavuckij, Mariya Bachmaha, Viatcheslav Krasiuk, Natasha Rybak, Timothy P. Flanigan, Vasyl Petrenko, Megan B. Murray

**Affiliations:** 1 Division of General Internal Medicine, The University of Texas Health Science Center at Houston - McGovern Medical School, Houston, TX, USA; 2 World Health Organization, Illiyinskaya Street, Kyiv, Ukraine; 3 Brown University School of Public Health, Providence, RI, USA; 4 Department of Pulmonology, Bogomolets National Medical University, Kyiv, Ukraine; 5 Division of Infectious Diseases, The Miriam Hospital, Warren Alpert Medical School at Brown University, Providence, RI, USA; 6 Department of Global Health and Social Medicine, Harvard Medical School, Boston, MA, USA

**Keywords:** tuberculosis, drug sensitive, treatment outcomes, patient predictors, HIV, HIV-TB coinfection, Ukraine, Eastern Europe

## Abstract

**Background**: Ukraine has high rates of poor treatment outcomes among drug sensitive tuberculosis (DSTB) patients, while global treatment success rates for DSTB remain high.

We evaluated baseline patient factors as predictors of poor DSTB treatment outcomes.

**Methods**: We conducted a retrospective analysis of new drug sensitive pulmonary TB patients treated in Kyiv Oblast, Ukraine between November 2012 and October 2014. We defined good treatment outcomes as cure or completion and poor outcomes as death, default (lost to follow up) or treatment failure. We performed logistic regression analyses, using routine program data, to identify baseline patient factors associated with poor outcomes.

**Results:** Among 302 patients, 193 (63.9%) experienced good treatment outcomes while 39 (12.9%) failed treatment, 34 (11.3%) died, and 30 (9.9%) were lost to follow up. In the multivariate analysis, HIV positive patients on anti-retroviral therapy (ART) [OR 3.50; 95% CI 1.46 – 8.42; p 0.005] or without ART (OR 4.12; 95% CI 1.36 – 12.43; p 0.01) were at increased risk of poor outcomes. Smear positivity (OR 1.75; 95% CI 1.03 - 2.97; p 0.04) was also associated with poor treatment outcomes.

**Conclusions:** High rates of poor outcomes among patients with newly diagnosed drug sensitive TB in Kyiv Oblast, Ukraine highlight the urgent need for programmatic interventions, especially aimed at patients with the highest risk of poor outcomes.

## Introduction

Tuberculosis (TB) control remains challenging worldwide, with approximately 10 million new cases diagnosed and 1.5 million TB deaths in 2018 ^1^. Although incidence rates have declined in parts of Eastern Europe, TB continues to be a significant public health problem in many former Soviet Union countries including Ukraine, which currently has the second highest burden of multi-drug resistant TB (MDR-TB) in the WHO European Region after Russia ^1^. National TB control measures in Ukraine include annual screening with chest radiographs for at risk groups i.e. immunosuppressed patients, diabetics, homeless patients, migrants, incarcerated individuals, and all medical staff in primary healthcare facilities ^2^. In addition, surveillance for resistant TB includes routine drug susceptibility testing (DST) for all culture positive isolates; and in 2007 the country adopted WHO recommended directly observed therapy short course (DOTS). However, despite these efforts, Ukraine’s National TB Program (UNTP) still has low treatment success rates. According to the most recent WHO data available for new smear and/or culture positive TB cases in Ukraine, the treatment success rate among these cases was 58% in 2011 in contrast to a global success rate of 85% ^3^.

TB treatment outcomes vary depending on the distribution of risk factors within a treatment cohort, as well as on the quality and nature of TB health services. Patient-related predictors of poor outcome among patients with drug-sensitive TB (DSTB) include gender, HIV, diabetes mellitus (DM), alcohol or substance use disorder, and homelessness ^4– 21^. Health system factors that influence TB outcomes include ease of access to services, diagnostic capabilities, drug availability, social support for patients, duration of hospitalization and collaboration of TB/HIV services ^11, 22, 23^. Currently, no published research addresses predictors of poor DSTB outcomes in the context of the UNTP, despite the frequency of this outcome. Here, we used routinely collected program data in the Kyiv Oblast of Ukraine to examine the association between baseline patient risk factors and DSTB treatment outcomes. These findings can help develop targeted interventions to address patient populations at the greatest risk of poor outcomes.

## Materials and methods

### Ethical statement

The study was approved by the Institutional Review Board at The Miriam Hospital, Lifespan, Providence; RI (215014 45CFR 46.110[5]) and the Research Ethics Committee at Bogomolets Medical University in Kyiv, Ukraine. Informed consent was not required because the data were analyzed anonymously, and written informed consent was waived by the Institutional Review Boards.

### Setting and study design

We conducted a retrospective chart review to identify baseline risk factors for poor treatment outcomes among drug-sensitive pulmonary TB patients in the Kyiv Oblast of Ukraine, where the notification rate for new pulmonary TB in 2014 was approximately 62 per 100 000 persons. TB diagnosis and management in Kyiv Oblast is provided free of charge and according to Ukraine’s NTP ^2^. National guidelines specify that all patients in need of evaluation for TB undergo sputum smear microscopy and culture, molecular testing with Xpert ^®^ MTB/RIF and chest X-ray to confirm diagnosis. Individuals in need of evaluation for TB include patients evaluated in primary care settings with complaints of cough, fever, night sweats, weight loss, chest pain, and dyspnea or patients that providers consider at risk for TB based on clinical history. General practitioners then refer such patients to TB specialists for diagnosis and further management. The 2014 UNTP specify the following: baseline susceptibility testing to rifampin (R), isoniazid (H), ethambutol (E), pyrazinamide (Z) and streptomycin (S) on all culture positive TB isolates; baseline screening for pre-specified risk factors including frequent alcohol use, intravenous drug use (IVDU), and homelessness (notably, screening for frequent alcohol use and substance use relies on patient self-report without specific definitions about what is considered high or harmful alcohol consumption); baseline HIV testing and provision of anti-retroviral therapy (ART) to those that are positive as soon as possible after initiation of TB treatment; and repeat DST among DSTB patients who are culture positive at three months or at the end of treatment. In the Kyiv Oblast laboratory, solid and liquid culture DST for first line drugs are performed using LJ medium and the M960 system (Becton Dickinson Microbiology System, Sparks, NV, USA). In Kyiv Oblast, treatment for alcohol or substance use disorder is not provided for patients during TB treatment.

UNTP guidelines also specify that DSTB patients receive treatment with two months of RHZE and four months of RH. The previous UNTP guidelines specified inpatient treatment during the intensive phase in specialized TB hospitals, while the subsequent continuation phase occurs in an ambulatory setting. Dedicated adult TB hospitals exist in each administrative region of Ukraine where patients receive testing and treatment. Although latest national guidelines in 2014 now recommend outpatient management of DSTB ^2^, many regions in Ukraine, including Kyiv Oblast, have yet to implement this practice and continue to hospitalize patients during the intensive phase.

For newly diagnosed patients with HIV, ART is initiated during hospitalization and after discharge, HIV-related care occurs at HIV programs that are distinct from the TB clinics, which provide outpatient TB care. Standard ART regimen for co-infected patients include Tenofovir, Lamivudine, and Efavirenz. In Kyiv Oblast, outpatients may receive daily directly observed therapy or receive a supply of medication at 7 – 10 day intervals. Clinicians can continue the inpatient care of individuals at high risk for lost to follow up (e.g. homeless patients) for the entire treatment duration, although compliance is not enforced; and patients are free to leave the hospital any time. UNTP guidelines also recommend follow up for DSTB patients at yearly intervals for three years after treatment completion.

### Data collection and statistical analysis

We analyzed routinely collected clinical and programmatic data from the three TB hospitals in Kyiv Oblast, which together admit approximately 1100 patients annually for pulmonary TB. We have previously reported on a cohort of approximately 600 patients initiated on treatment for drug resistant TB in Kyiv Oblast between 2012 and 2015 ^24^. For this study, we included all adult patients (≥ 16 years) treated for newly diagnosed drug-sensitive pulmonary TB between November 2012 and October 2014. We excluded patients who did not yet have a treatment outcome assigned because they were undergoing the initial course of TB treatment at the time of data extraction in November 2014, and those with previous TB history.

We extracted the following information routinely collected by the TB program in an electronic database: age, gender, residence, employment status, history of TB contact, homelessness, immigration status, previous incarceration, HIV status with ART initiation dates, history of frequent alcohol use and intravenous (IV) drug use, as well as mode of case finding (active or passive); passive TB case finding refers to the diagnosis of TB among patients who self-initiate contact with healthcare providers for management of TB symptoms. We also recorded baseline sputum smear, culture and DST results. The NTP provides standardized paper forms used by TB providers to record all baseline demographic and clinical information for routine program monitoring. In Kyiv Oblast TB hospitals, all data are subsequently entered in an electronic database by the statistics department.

Treatment outcomes for DSTB are classified in Kyiv Oblast according to WHO guidelines ^25^. Good treatment outcomes include cure and treatment completion, while poor outcomes include deaths, lost to follow up and treatment failure. The WHO considers a DSTB patient cured if he or she remains smear or culture negative in the last month of treatment and on at least one previous occasion. A patient is considered to have completed treatment if he or she has received a full course of therapy but has not received smear or culture in the last month of treatment. Any deaths during TB treatment are considered TB related. A patient is considered to be lost to follow up if he or she interrupts treatment for two or more consecutive months. Patients who remain smear or culture positive at month 5 or later during treatment are considered treatment failures; and in Kyiv Oblast patients who acquire resistance are also categorized as treatment failures. The exact dates of treatment outcomes or last follow up visits were not captured in the database.

We analyzed only patients with confirmed drug sensitive pulmonary TB, and we excluded from the main analysis patients who transferred out or had missing outcomes. We did not follow up with patients in the community to ascertain treatment outcome among those with missing data on final outcome. We compared categorical variables with Fisher’s exact test and continuous variables with the Wilcoxon rank sum test. We performed univariate and multivariate logistic regression analyses to identify baseline predictors of combined poor treatment outcomes (death, failure, and lost to follow up). For the multivariate model, we included baseline variables previously known to be associated with poor outcomes (age, sex, HIV, high alcohol consumption, homelessness) and any variable associated with poor outcomes at p value less than 0.2 in the univariate analysis. We further evaluated baseline predictors for the outcomes of death and treatment failure separately. We used complete case analysis in the regression models. We used the regression coefficients specified by the final multivariate model to predict probability of combined poor outcomes. In a sensitivity analysis, we categorized patients whose treatment outcomes were not assessed (transferred out and missing final outcome data) as having poor outcomes. Data were analyzed using SAS v9.4 (SAS Institute, Cary, NC 2013).

## Results

**Figure 1.**
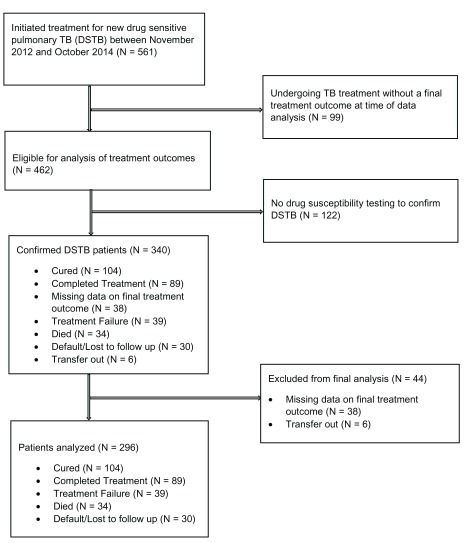
Flow diagram of drug sensitive pulmonary TB cases analyzed.

We identified 561 patients treated for new DSTB between November 2012 and October 2014. Among them, we excluded 99 (17.6%) patients who did not yet have a treatment outcome because they were still undergoing TB treatment at the time of analysis ( Figure 1). Table 1 lists baseline characteristics of the remaining 462 patients; among them, 122 (26.4%) patients had no drug susceptibility testing performed. Three hundred and forty patients (73.6%) had a baseline DST to confirm drug sensitive pulmonary TB, and 181 (39.2%) underwent Xpert/Rif testing at baseline. Seventy-five (16.2%) patients tested HIV positive, while HIV status was not recorded for 8 (1.7%) patients. Among the HIV positive patients, 34 (45.3%) were initiated on ART during TB treatment. Median time to ART initiation from TB treatment start date was 43.5 days (IQR 34.0 – 59.5).

**Table 1.** Baseline characteristics of drug-sensitive tuberculosis patients in Kyiv Oblast (November 2012 to October 2014, N = 462). IQR: Interquartile Range.

	N (%) or median (IQR)
Age	40.0 (33.0 – 52.0)
Male	351 (76.0)
HIV status	
Negative	379 (82.0)
Positive	75 (16.2)
Unknown	8 (1.7)
Initiated on ART among HIV positive	34 (45.3)
Median days to ART initiation	43.5 (34.0 – 59.5)
Rural residence ^a^	246 (58.9)
Frequent alcohol use	69 (14.9)
Intravenous drug use (IVDU)	6 (1.3)
Known TB contact at diagnosis	4 (0.9)
Homeless	11 (2.4)
Unemployed	256 (55.4)
Migrant from outside Kyiv Oblast	1 (0.2)
Previous Incarceration	7 (1.5)
Passive Case finding ^b^	377 (81.9)
Smear Positive at baseline ^c^	242 (52.6)
Have baseline Drug Susceptibility Test (DST) result	340 (73.6)
Have baseline Xpert/RIF Result	181 (39.2)

^a^ N = 418 ^b^ N = 461 ^c^ N = 460

Of the 340 patients with DST results, 38 (11.2%) had missing outcome data. Among the remaining 302 patients, 104 (34.4%) experienced treatment cure and 89 (29.5%) completed treatment, while 39 (12.9%) failed treatment, 34 (11.3%) died, 30 (9.9%) were lost to follow up, and 6 (2.0%) transferred out.

In the univariate analysis, significant baseline predictors of poor treatment outcomes included frequent alcohol use (OR 1.95; 95% CI 1.05 - 3.61; p 0.03), and smear positive disease (OR 1.70; 95% CI 1.04 - 2.75; p 0.03) ( Table 2). Compared to HIV negative, HIV patients were also at increased risk of poor outcomes; those who were not initiated on ART were four times as likely to experience poor outcomes (OR 4.07; 95% CI 1.45 – 11.39; p 0.01), while patients on ART were more than twice as likely to have a poor treatment outcome (OR 2.58; 95% CI 1.14 – 5.85; p 0.02). Homeless patients were also at increased risk of poor outcomes, although this association was not significant at the .05 level (OR 7.76; 95% CI 0.86 – 70.32). Unemployment (OR 1.59; 95% CI 0.97 – 2.61; p 0.06) and passive case finding (OR 1.78; 95% CI 0.94 – 3.39; p 0.07) also conferred borderline significantly increased risk of poor treatment outcomes in the univariate analysis. Time to ART initiation was not associated with poor outcomes (OR 1.02; 95% CI 0.98 – 1.06; p 0.33) ( Table 2).

**Table 2.** Univariate and multivariate analyses of baseline predictors of combined poor outcomes among drug-sensitive TB patients. OR: Odds Ratio.

	Univariate Model (N = 296) ^a^		Multivariate Model (N = 292)	
	OR (95% CI)	p value	Adjusted Odds Ratio ^b^ (95% CI)	p value
Age	1.01 (0.99 – 1.02)	0.48	1.01 (0.99 – 1.03)	0.18
Male	1.12 (0.63 – 1.99)	0.70	1.24 (0.66 – 2.34)	0.50
HIV negative ^c^	Ref		Ref	
HIV positive with ART	2.58 (1.14 – 5.85)	0.02	3.50 (1.46 – 8.42)	0.005
HIV positive without ART	4.07 (1.45 – 11.39)	0.01	4.12 (1.36 – 12.43)	0.01
Time to ART initiation ^d^	1.02 (0.98 – 1.06)	0.33	NA	
Frequent alcohol use	1.95 (1.05 – 3.61)	0.03	1.81 (0.93 – 3.55)	0.08
Homeless	7.76 (0.86 – 70.32)	0.07	6.38 (0.69 – 59.40)	0.10
Smear positive ^c^	1.70 (1.04 – 2.75)	0.03	1.75 (1.03 – 2.97)	0.04
Rural ^e^	1.23 (0.73 – 2.07)	0.42	NA	
Unemployed	1.59 (0.97 – 2.61)	0.06	1.26 (0.72 – 2.20)	0.43
TB contact	5.76 (0.59 – 56.05)	0.13	NA ^f^	
Passive case finding	1.78 (0.94 – 3.39)	0.07	1.18 (0.60 – 2.35)	0.63

^a^ Patients with confirmed drug sensitive TB and outcomes of cure, completion, death, treatment failure and default. ^b^ Adjusted for age, gender, HIV, frequent alcohol use, homelessness, baseline smear status, unemployment and passive case finding. ^c^ N = 294 ^d^ N = 26 ^e^ N = 269 ^f^ Excluded from multivariate analysis because only 4 patients had known TB contact.

When we adjusted for other risk factors, we found that smear positivity (OR 1.75; 95% CI 1.03 - 2.97; p 0.04) and HIV positivity (on ART [OR 3.50; 95% CI 1.46 – 8.42; p 0.005] and without ART [OR 4.12; 95% CI 1.36 – 12.43; p 0.01]) all remained significant predictors of poor outcome. Patients with frequent alcohol use also had a modest increase in risk of poor outcomes (OR 1.81; 95% CI 0.93 – 3.55; p 0.08) and the odds of poor outcomes among the homeless continued to be high but not statistically significant at 6.38 (95% CI 0.69 – 59.40) ( Table 2). Unemployment (OR 1.26; 95% CI 0.72 – 2.20; p 0.43) and passive case finding (OR 1.18; 95% CI 0.60 – 2.35; p 0.63) were no longer associated with increased risk of poor outcomes in the adjusted analysis ( Table 2). Our multivariate model predicted that a 40-year-old male who is HIV positive but not on ART, with frequent alcohol use and smear positive disease, has a 75.8% probability of poor treatment outcome.

When we separately evaluated risk factors for death during DSTB treatment, in the adjusted analysis, we found age (OR 1.03; 95% CI 1.00 – 1.06; p 0.03), HIV positivity (OR 4.21; 95% CI 1.44 – 12.30; p 0.01) and frequent alcohol use (OR 2.54; 95% CI 1.00 – 6.42; p 0.05) were associated with statistically significant increased risk of death ( Table 3). HIV positivity (OR 7.42; 95% CI 2.56 – 21.54; p < 0.001) and smear positive disease at baseline (OR 4.99; 95% CI 2.00 – 12.45; p 0.001) were the strongest predictors of DSTB treatment failure ( Table 4).

**Table 3.** Univariate and multivariate analyses of baseline predictors of death among drug-sensitive TB patients. OR: Odds Ratio.

	Univariate Model (N = 227) ^a^		Multivariate Model (N = 224)	
	OR (95% CI)	p value	Adjusted Odds Ratio ^b^ (95% CI)	p value
Age	1.02 (1.00 – 1.05)	0.05	1.03 (1.00 – 1.06)	0.03
Male	0.85 (0.37 – 1.94)	0.69	1.05 (0.42 – 2.63)	0.91
HIV positive ^c^	2.96 (1.17 – 7.49)	0.02	4.21 (1.44 – 12.30)	0.01
Frequent alcohol use	2.31 (0.97 – 5.50)	0.06	2.54 (1.00 – 6.42)	0.05
Homeless	12.00 (1.06 – 136.23)	0.05	NA ^d^	
Smear positive ^e^	1.84 (0.88 – 3.85)	0.11	1.80 (0.81 – 3.98)	0.15
Rural ^f^	1.35 (0.60 – 3.06)	0.47	NA	
Unemployed	1.38 (0.66 – 2.92)	0.40	NA	
TB contact	5.82 (0.36 – 95.42)	0.22	NA	
Passive case finding	10.03 (1.34 – 75.43)	0.03	7.04 (0.91 – 54.15)	0.06

^a^ Patients with confirmed drug sensitive TB and outcomes of cure, completion and death. ^b^ Adjusted for age, gender, HIV, frequent alcohol use, baseline smear status, and passive case finding. ^c^ N = 225 ^d^ Excluded from multivariate analysis because there were only 3 homeless patients. ^e^ N = 226 ^f^ N = 206

**Table 4.** Univariate and multivariate analyses of baseline predictors of treatment failure among drug-sensitive TB patients. OR: Odds Ratio.

	Univariate Model (N = 232) ^a^		Multivariate Model (N = 228)	
	OR (95% CI)	p value	Adjusted Odds Ratio ^b^ (95% CI)	p value
Age	0.99 (0.97 – 1.02)	0.47	0.99 (0.96 – 1.03)	0.70
Male	2.07 (0.76 – 5.60)	0.15	2.27 (0.77 – 6.69)	0.14
HIV positive ^c^	4.27 (1.85 – 9.85)	0.001	7.42 (2.56 – 21.54)	<0.001
Frequent alcohol use	0.95 (0.34 – 2.63)	0.91	0.90 (0.30 – 2.72)	0.86
Homeless	10.38 (0.92 – 117.41)	0.06	NA ^d^	
Smear positive ^c^	2.79 (1.33 – 5.85)	0.01	4.99 (2.00 – 12.45)	0.001
Rural ^e^	0.90 (0.43 – 1.88)	0.78	NA	
Unemployed	1.37 (0.68 – 2.77)	0.38	NA	
TB contact	10.38 (0.92 – 117.41)	0.06	NA ^d^	
Passive case finding	2.07 (0.76 – 5.60)	0.15	1.44 (0.50 – 4.13)	0.50

^a^ Patients with confirmed drug sensitive TB and outcomes of cure, completion and treatment failure. ^b^ Adjusted for age, gender, HIV, frequent alcohol use, baseline smear status, and passive case finding. ^c^ N = 230 ^d^ Excluded from multivariate analysis because there were only 3 homeless patients and 3 patients with known TB contact. ^e^ N = 211

When we categorized patients with missing outcome data and patients who transferred out as having poor outcomes, the results did not differ from the findings in our main analysis of predictors of poor treatment outcomes ( Table 5).

**Table 5.** Sensitivity univariate and multivariate analyses of baseline predictors of poor outcomes among drug-sensitive TB patients. ^a^ OR: Odds Ratio

	Univariate Model (N = 340)		Multivariate Model (N = 333)	
	OR (95% CI)	p value	Adjusted Odds Ratio ^b^ (95% CI)	p value
Age	1.01 (0.99 – 1.02)	0.48	1.01 (0.99 – 1.02)	0.35
Male	1.09 (0.65 – 1.83)	0.74	1.19 (0.66 – 2.07)	0.54
HIV negative ^c^	Ref		Ref	
HIV positive with ART	2.30 (1.07 – 4.95)	0.03	2.84 (1.28 – 6.33)	0.01
HIV positive without ART	3.57 (1.33 – 9.57)	0.01	3.27 (1.16 – 9.24)	0.03
Time to ART initiation ^d^	1.02 (0.98 – 1.07)	0.30	NA	
Frequent alcohol use	1.72 (0.97 - 3.04)	0.06	1.60 (0.88 – 2.91)	0.12
Homeless	6.76 (0.78 – 58.49)	0.08	5.44 (0.61 – 48.41)	0.13
Smear positive ^e^	1.60 (1.04 - 2.47)	0.03	1.55 (0.98 – 2.47)	0.06
Rural ^f^	1.17 (0.74 – 1.86)	0.50	NA	
Unemployed	1.21 (0.78 – 1.86)	0.40	NA	
TB contact	3.99 (0.41 – 38.77)	0.23	NA ^h^	
Passive case finding ^g^	1.55 (0.89 – 2.68)	0.12	1.13 (0.62 – 2.04)	0.69

^a^ Patients with confirmed drug sensitive TB and poor outcomes defined as death, treatment failure, loss to follow up, transferred out, and missing outcome data. ^b^ Adjusted for age, gender, HIV, frequent alcohol use, homelessness, baseline smear status, and passive case finding. ^c^ N = 336 ^d^ N = 29 ^e^ N = 338 ^f^ N = 310 ^g^ N = 339 ^h^ Excluded from multivariate analysis because only 4 patients had known TB contact.

Data for patients analyzed in the retrospective cohort study Click here for additional data file. Copyright: © 2019 Aibana O et al. 2019 Data associated with the article are available under the terms of the Creative Commons Zero "No rights reserved" data waiver (CC0 1.0 Public domain dedication).

## Discussion

We found that only 64% of patients treated for drug-sensitive TB in Kyiv Oblast achieved treatment cure or completion, and this is far below global treatment success rates of 85% ^1^. We also identified frequent alcohol use and HIV as patient determinants of failure, death or lost to follow up in this setting. Our findings support the idea that TB control efforts in this setting should urgently prioritize interventions aimed at the patient populations identified as at risk.

We show that routinely collected baseline programmatic data in Ukraine’s NTP reasonably predicts patients at high risk of poor DSTB treatment outcome at the beginning of treatment. Notably, this routine program data did not include other known predictors of poor TB treatment outcomes (e.g. DM, smoking, socioeconomic status, and poor nutritional status) ^4, 13, 15, 20, 21, 26– 29^, therefore, we could not evaluate relative contributions of these unmeasured patient factors. Furthermore, the UNTP does not employ validated screening tools for harmful alcohol or substance use but instead relies on patient self-report; stigma associated with high alcohol consumption and IVDU likely limits patients’ willingness to accurately report this information. Hence, rates of reported alcohol and IVDU were likely underestimated. Studies from other settings have demonstrated that incorporating dedicated treatment for high alcohol use within TB programs is feasible ^30^ and access to treatment for substance use improves TB outcomes ^31, 32^. For instance, one study in Ukraine showed methadone treatment for TB patients with IVDU led to improved retention in care and medication adherence ^32^. Nevertheless, despite the limitations of routine program data, our findings demonstrate that within the current operations of Ukraine’s TB program, there is sufficient data to identify patients who can be targeted for early intervention to mitigate their risk of poor outcome. Improved screening for additional co-morbidities will also help identify other populations at higher risk for poor TB outcomes in this setting.

It is also important to note that health system factors influence patient-predictors and limit treatment success rates among patients with and without known risk factors at baseline. Hence, TB control efforts in this setting should also address how the current TB care delivery system in Ukraine adversely affects treatment outcomes for all patients. For instance, while the TB program in Kyiv Oblast tested most patients for HIV, less than half of HIV/TB co-infected patients were initiated on ART. We do not have data on the specific reasons why many HIV-positive patients were not initiated on ART during TB treatment. Anecdotal reports indicate TB providers sometimes defer ART for patients with high CD4 counts despite national guidelines for ART initiation regardless of CD4 count. Even among those treated, ART did not significantly mitigate the risk of poor TB treatment outcomes. Timing of ART initiation also did not predict outcome. Health system factors such as quality of ART and inconsistent availability may explain these findings. Provision of ART to all HIV/TB patients, as recommended by the WHO ^33^, will likely improve treatment success rates among co-infected patients. However, additional system interventions such as integrated HIV and TB care during the entire phase of TB treatment may also be required to sufficiently address the excess risk of poor TB outcomes among HIV patients in this setting.

We found that the majority of TB patients were identified through passive case finding, which may also contribute to poor treatment outcomes. The WHO recommends systematic screening for active TB as a complement to passive case finding ^34^. Studies have also shown that active TB case finding results in early detection and reduces risk of extensive disease at diagnosis ^35– 39^, which may potentially decrease risk of poor outcomes. Active case finding also reduces risk of TB transmission ^39– 42^ and may contribute to reducing TB prevalence. Lack of active case TB finding may further result in under notification of TB in this setting. TB control efforts in Ukraine will likely benefit from strengthening and improving health systems capacity for active case finding. The use of newer technologies and approaches to optimize early identification of TB patients and prompt diagnosis of resistant TB may also lead to improved treatment outcomes in this setting.

Our finding that 10% of patients are lost to follow up also highlight the importance of providing patient support to all TB patients during treatment, not just individuals identified at baseline as having high risk for poor outcomes. Prior studies have shown incentives and other enablers of treatment adherence are an effective strategy for improving TB treatment outcomes ^43– 47^. Such enablers include use of community health workers, food and transportation assistance, reminder systems, education and counseling geared towards adherence as well as enhanced access to social services. Although Ukraine’s newest TB guidelines encourage additional social support for TB patients, currently there is limited funding dedicated to incentivizing treatment adherence.

Our study is limited by use of routine programmatic data, which did not include assessment of other known predictors of DSTB outcomes. We also found a high proportion of patients did not have their treatment outcomes assessed which further reduces the rate of successful treatment outcomes in this setting. However, when we included patients with missing data on final outcomes in the analysis, our results did not change; we identified the same patient predictors of poor treatment outcomes.

Previous evaluations of Ukraine’s TB program have already enumerated specific health system factors that hamper successful treatment outcomes, including: unnecessarily prolonged hospital-based care; interruptions in drug supply; protocol deviations; limited social support for patients; and suboptimal infection prevention that increases nosocomial TB transmission ^48, 49^. Our findings have been presented to policy makers in Ukraine including during a National Round Table discussion in Kyiv (November 2015) in preparation for updates to UNTP guidelines, which will focus on scaling up ambulatory-based care, targeted interventions for populations at risk of poor outcomes and patient-oriented approaches to improve treatment adherence. New policy changes create the possibility of further analyzing health system contributions to poor outcomes, and assessing how systems improvements will influence success rates among patients with baseline increased risk of poor outcomes. Future research can also evaluate providers’ understanding of and compliance with guidelines.

## Conclusion

We found extremely low rates of treatment cure and completion for new drug sensitive TB in the Kyiv Oblast of Ukraine. In addition to specific interventions targeted at vulnerable patients, there is also a need to address and mitigate the impact of health system factors on Ukraine’s TB treatment success rates.

## Data availability

The data referenced by this article are under copyright with the following copyright statement: Copyright: © 2019 Aibana O et al.

Data associated with the article are available under the terms of the Creative Commons Zero "No rights reserved" data waiver (CC0 1.0 Public domain dedication). 

Data have been de-identified for ethical, data protection and security reasons. Permission for use and publication of the anonymized data granted by the Institutional Review Board of Bogomolets Medical University.

**Dataset 1. Data for patients analyzed in the retrospective cohort study.** DOI, 10.5256/f1000research.12687.d179513 ^50^.
