# Supplementary material for: Patient predictors of poor drug sensitive tuberculosis treatment outcomes in Kyiv Oblast, Ukraine
Source: F1000Res. 2019 Nov 22;6:1873. Originally published 2017 Oct 23. [Version 3] doi: 10.12688/f1000research.12687.3 (PMC6859782; doi:10.12688/f1000research.12687.3)
Supplement: Peer review history

# Peer review: Reviewer response for version 2

**DOI:** 10.5256/f1000research.15502.r56295

**Published:** 2019-11-15

**Authors:** Samantha Flores-Treviǹo

**Affiliations:** 1 Autonomous University of Nuevo León, Monterrey, Mexico

The work of Aibana et al. is highly relevant regarding TB treatment outcomes in Ukraine. The authors have made an excellent improvement considering all previous corrections made by the reviewers. The article is well-written and the results are critical for the guidelines of the national TB program. 

 Nevertheless, I have some very minor revisions that can be easily amended. I think these corrections would improve the quality of the manuscript. 

 Abstract: 

 In the results section, you added "Frequent alcohol use (OR 1.81; 95% CI 0.93 - 3.55; p 0.08) and smear positivity (OR 1.75; 95% CI 1.03 - 2.97; p 0.04) were also associated with poor treatment outcomes." However, the p value of alcohol use was 0.08, and cannot be considered to be statistically significant. I would rather change the sentence to: "Smear positivity (OR 1.75; 95% CI 1.03 - 2.97; p 0.04) was also associated with poor treatment outcomes."

 Methods:

 M960 system should also include: (Becton Dickinson Microbiology System, Sparks, NV, USA) if it is the case. 

 "For the multivariate model, we included baseline variables previously known to be associated with poor outcomes (age, sex, HIV, high alcohol consumption, homelessness) and any variable associated with poor outcomes at p value less than 0.2 in the univariate analysis." Should it not be less than 0.5?

 Results:

 In the first paragraph two sentences (340 and 75) start with a number. You spell out numbers at the start of a sentence. 

 Discussion:

 "We found that majority of TB patients were identified through passive case finding, which may also contribute to poor treatment outcomes." change to "We found that the majority of TB patients were identified through passive case finding, which may also contribute to poor treatment outcomes." 

 References:

 I think outdated references such as Schluger *et al*, 1995 and Volmink and Garner, 1997, should be deleted.

I confirm that I have read this submission and believe that I have an appropriate level of expertise to confirm that it is of an acceptable scientific standard.

# Peer review: Reviewer response for version 2

**DOI:** 10.5256/f1000research.15502.r31869

**Published:** 2018-03-13

**Authors:** Bernt Lindtjørn

**Affiliations:** 1 Center for International Health, University of Bergen, Bergen, Norway

The authors have addressed the issues I raised.

I confirm that I have read this submission and believe that I have an appropriate level of expertise to confirm that it is of an acceptable scientific standard.

# Peer review: Reviewer response for version 1

**DOI:** 10.5256/f1000research.13738.r29231

**Published:** 2018-02-05

**Authors:** Erica Lessem

**Affiliations:** 1 Treatment Action Group, New York, NY, USA

This is an important article, highlighting the previously overlooked problem of poor outcomes in nearly half of people with drug-susceptible TB, which is usually considered "low hanging fruit" and relatively easy to treat (as opposed to more complicated drug-resistant TB). The article is generally well-written and provides important specifics to help readers understand what was done, the context, and what conclusions can be fairly drawn from the data. It is good that the authors highlight the importance of ART initiation and earlier ART, ambulatory care, screening for other known correlates of poor outcomes (DM, smoking, etc.), as well as employing validated screening tools for potential risk behaviors (alcohol and drug use)

 There are a few areas that could be improved:

- I was surprised that a major recommendation was not to conduct more active case finding. The article mentions briefly that there is some active case finding in this setting, but it is not analyzed that 82% of cases were identified through passive case finding. While not identified as a risk factor for poor DS-TB per this analysis, it could be just that there were so few cases for active case finding that the N in the comparison was too small for significance. The fact that the vast majority of cases are found through passive case finding indicates a weak system for active finding, which leads to late detection, higher probability of transmission to more people, and greater chance of extensive disease (which could contribute to poor outcomes).
- Relatedly, cavitation (which is a marker of extent of disease) was not explored as a risk factor for poor outcomes and could likely be easily included as part of a baseline analysis of risk if Ukraine is already routinely performing radiography on all patients.
- Aside from individual patient risk factors, the fact that 11% of cases are missing data and the high rate of loss to follow up indicate need for patient support across the board to ensure patients are able to stay in care through cure.
- The authors do not describe what kind of interventions could help improve outcomes for those at high risk, despite a large body of evidence on useful elements of patient support.
- Avoid the use of stigmatizing language, in line with recommended language: http://www.stoptb.org/assets/documents/resources/publications/acsm/LanguageGuide_ForWeb20131110.pdf. For example, use "lost to follow up" as opposed to "defaulted"; "persons in need of evaluation for TB" instead of "suspects" and define what is considered "alcohol abuse" and consider using a less stigmatizing term (which is indeed avoided in certain parts of the document) such as "frequent alcohol use" or "high alcohol consumption").
- While not the main focus of the article, the authors refer to but do not make notes about several areas where the UNTP guidance is inappropriate; it is worth calling these out as well. For example:
   
  - The authors note that routine DST is done for all culture positive cases but do not specify using what method-- liquid or solid culture phenotypic testing? Are line probe assays available? The availability (or lack thereof) of rapid methods of DST (in addition to Xpert, which only identifies rifampicin resistance) could be another recommendation that could help improve treatment outcomes by identifying need for regimen changes earlier on.
  - Chest radiography is done for high risk groups annually --this is potentially a lot of exposure to radiation—why not use symptom screen and Xpert?

I confirm that I have read this submission and believe that I have an appropriate level of expertise to confirm that it is of an acceptable scientific standard, however I have significant reservations, as outlined above.

# Response

**Published:** 2018-03-06

**Authors:** Omowunmi Aibana

**Affiliations:** University of Texas Health Science Center at Houston, USA

**Comment 1:** I was surprised that a major recommendation was not to conduct more active case finding. The article mentions briefly that there is some active case finding in this setting, but it is not analyzed that 82% of cases were identified through passive case finding. While not identified as a risk factor for poor DS-TB per this analysis, it could be just that there were so few cases for active case finding that the N in the comparison was too small for significance. The fact that the vast majority of cases are found through passive case finding indicates a weak system for active finding, which leads to late detection, higher probability of transmission to more people, and greater chance of extensive disease (which could contribute to poor outcomes).

**Response 1: We have added the following to our discussion (paragraph 4):**

**“We found that majority of TB patients were identified through passive case finding, which may also contribute to poor treatment outcomes. The WHO recommends systematic screening for active TB as a complement to passive case finding [1]. Studies have also shown that active TB case finding results in early detection and reduces risk of extensive disease at diagnosis [2-6], which may potentially decrease risk of poor outcomes. Active case finding also reduces risk of TB transmission [6-9] and may contribute to reducing TB prevalence.** **Lack of active case TB finding may further result in under notification of TB in this setting.** **TB control efforts in Ukraine will likely benefit from strengthening and improving health systems capacity for active case finding.”**

1. World Health Organization. Systematic screening for active tuberculosis. Principles and Recommendations. 2013. Available: http://apps.who.int/iris/bitstream/10665/84971/1/9789241548601_eng.pdf?ua=1 Accessed 20 February 2018.    

2. den Boon S, Verver S, Lombard CJ, Bateman ED, Irusen EM, Enarson DA, et al. Comparison of symptoms and treatment outcomes between actively and passively detected tuberculosis cases: the additional value of active case finding. Epidemiol Infect. 2008;136(10): 1342-1349. 

3. Eang MT, Satha P, Yadav RP, Morishita F, Nishikiori N, van-Maaren P, et al. Early detection of tuberculosis through community-based active case finding in Cambodia. BMC Public Health. 2012;12: 469.

4. Datiko DG, Yassin MA, Theobald SJ, Blok L, Suvanand S, Creswell J, Cuevas LE. Health extension workers improve tuberculosis case finding and treatment outcome in Ethiopia: a large-scale implementation study. BMJ Glob Health. 2017;2(4): e000390.

5. Kranzer K, Afnan-Holmes H, Tomlin K, Golub JE, Shapiro AE, Schaap A, Corbett EL, Lönnroth K, Glynn JR. The benefits to communities and individuals of screening for active tuberculosis disease: a systematic review. Int J Tuberc Lung Dis. 2013 Apr;17(4):432-46.

6. Cheng S, Chen W, Yang Y, Chu P, Liu X, Zhao M, et al. Effect of diagnostic and treatment delay on the risk of tuberculosis transmission in Shenzhen, China: an observational cohort study, 1993-2010. PLoS One. 2013;8(6): e67516.

7. Lin X, Chongsuvivatwong V, Lin L, Geater A, Lijuan R. Dose-response relationship between treatment delay of smear-positive tuberculosis patients and intra-household transmission: a cross-sectional study. Trans R Soc Trop Med Hyg. 2008;102(8): 797-804.

8. Fox GJ, Barry SE, Britton WJ, Marks GB. Contact investigation for tuberculosis: a systematic review and meta-analysis. Eur Respir J. 2013;41(1): 140–156.

9. Jia ZCS, Ma Y, Zhang Y, Bai L, Xu W, He X, et al. Tuberculosis burden in China: a high prevalence of pulmonary tuberculosis in household contacts with and without symptoms. BMC Infect Dis. 2014;14: 64.

**Comment 2:** Relatedly, cavitation (which is a marker of extent of disease) was not explored as a risk factor for poor outcomes and could likely be easily included as part of a baseline analysis of risk if Ukraine is already routinely performing radiography on all patients.

**Response 2: Our analysis was based on information captured in the electronic database, which in Kyiv Oblast did not include an adequate level of detail regarding cavitary lesions. Therefore, we were unable to analyze cavitation as a predictor of poor outcomes in this study.**

**Comment 3:** Aside from individual patient risk factors, the fact that 11% of cases are missing data and the high rate of loss to follow up indicate need for patient support across the board to ensure patients are able to stay in care through cure.

The authors do not describe what kind of interventions could help improve outcomes for those at high risk, despite a large body of evidence on useful elements of patient support.

**Response 3: We have expanded our discussion (paragraphs 2 and 5) as follows:**

**“Studies from other settings have demonstrated that incorporating dedicated treatment for high alcohol use within TB programs is feasible [1] and access to treatment for substance use improves TB outcomes [2,3]. For instance, one study in Ukraine showed methadone treatment for TB patients with IVDU led to improved retention in care and medication adherence [3].”**

**“Our finding that 10% of patients are lost to follow up also highlights the importance of providing patient support to all TB patients during treatment, not just individuals identified at baseline as having high risk for poor outcomes. Prior** **studies have shown incentives and other enablers of treatment adherence are an effective strategy for improving TB treatment outcomes [4-8]. Such enablers include use of community health workers, food and transportation assistance, reminder systems, education and counseling geared towards adherence as well as enhanced access to social services. Although Ukraine’s newest TB guidelines encourage additional social support for TB patients, currently there is limited funding dedicated to incentivizing treatment adherence.”**

1 **. **Shin S, Livchits V, Connery H S,  Shields A, Yanov S, Yanova G, et al. Effectiveness of alcohol treatment interventions integrated into routine tuberculosis care in Tomsk, Russia. Addiction. 2013;108(8): 1387-1396.

2. Gelmanova IY, Taran DV, Mishustin SP, Golubkov AA, Solovyova AV, Keshavjee S. 'Sputnik':a programmatic approach to improve tuberculosis treatment adherence and 

outcome among defaulters. Int J Tuberc Lung Dis. 2011;15(10): 1373-1379.

3. Morozova, Dvoryak S, Altice FL. Methadone treatment improves tuberculosis treatment among hospitalized opioid dependent patients in Ukraine. Int J Drug Policy. 2013;24(6): e91-e98.

4. Schluger N, Ciotoli C, Cohen D, Johnson H, Rom WN. Comprehensive tuberculosis control for patients at high risk for noncompliance. Am J Respir Crit Care Med. 1995;151(5): 1486-90.

 5. Volmink J, Garner P. Systematic review of randomised controlled trials of strategies to promote adherence to tuberculosis treatment. BMJ. 1997; 315: 1403-1406.

 6. Davidson H, Schluger NW, Feldman PH, Valentine DP, Telzak EE, Laufer F N. The effects of increasing incentives on adherence to tuberculosis directly observed therapy. Int J Tuberc Lung Dis. 2000;4: 860-865.

 7. Liu Q, Abba K, Alejandria MM, Balanag VM, Berba RP, Lansang MA. Reminder systems and late patient tracers in the diagnosis and management of tuberculosis. Cochrane Database Syst Rev. 2008;4:  CD006594.

8. M'Imunya MJ, Kredo T, Volmink J. Patient education and counseling for promoting adherence to treatment for tuberculosis. Cochrane Database Syst Rev. 2012;5: CD006591.

Comment 4: Avoid the use of stigmatizing language, in line with recommended language: http://www.stoptb.org/assets/documents/resources/publications/acsm/LanguageGuide_ForWeb20131110.pdf. For example, use "lost to follow up" as opposed to "defaulted"; "persons in need of evaluation for TB" instead of "suspects" and define what is considered "alcohol abuse" and consider using a less stigmatizing term (which is indeed avoided in certain parts of the document) such as "frequent alcohol use" or "high alcohol consumption").

**Response 4: We have made changes throughout the manuscript to avoid stigmatizing language as suggested. We changed “default” to “lost to follow up,” replaced “alcohol abuse” with “frequent alcohol use” and now refer to TB suspects as individuals in need of evaluation for TB.**

**We have also clarified in the description of study setting (paragraph 1) that “** **screening for frequent alcohol use and substance use relies on patient self-report without specific definitions about what is considered high or harmful alcohol consumption.”** 

**Comment 5: **While not the main focus of the article, the authors refer to but do not make notes about several areas where the UNTP guidance is inappropriate; it is worth calling these out as well. For example:

The authors note that routine DST is done for all culture positive cases but do not specify using what method-- liquid or solid culture phenotypic testing? Are line probe assays available? The availability (or lack thereof) of rapid methods of DST (in addition to Xpert, which only identifies rifampicin resistance) could be another recommendation that could help improve treatment outcomes by identifying need for regimen changes earlier on.

Chest radiography is done for high risk groups annually -- this is potentially a lot of exposure to radiation—why not use symptom screen and Xpert?

**Response 5: We have now noted in the methods section that: “** **In the Kyiv Oblast laboratory, solid and liquid culture DST for first line drugs are performed using LJ medium and the M960 system.”**

**Please note that our analysis also revealed that available technology such as DST and Xpert that are currently mandated per UNTP guidelines remain under-utilized in Kyiv Oblast. For instance, less than half of patients in this cohort had a baseline Xpert test. Therefore, introduction of additional technology such as line probe assay, with its requisite costs in materials and training, may not necessarily have an immediate and beneficial impact on treatment outcomes in this setting.**

**Paragraph 4 in the discussion section now states: “The use of newer technologies and approaches to optimize early identification of TB patients and prompt diagnosis of resistant TB may also lead to improved treatment outcomes in this setting.”**

# Peer review: Reviewer response for version 1

**DOI:** 10.5256/f1000research.13738.r30175

**Published:** 2018-02-01

**Authors:** Bernt Lindtjørn

**Affiliations:** 1 Center for International Health, University of Bergen, Bergen, Norway

The authors address an important issue. I have the following comments:

 Most of the literature is old, and the authors should use more up-to-date literature. This also includes the methods and how to calculate default rates. The authors chose a method whereby they exclude patients who were lost to follow-up. However, this is a too simplistic approach. The authors should read the latest WHO recommendations on doing such calculations. 

 The biggest problem with this paper is how they handle missing information. They state that: "We analysed only patients with confirmed drug sensitive pulmonary TB, and we excluded from the analysis patients who transferred out or had missing outcomes."  Also, as there is a high prevalence of drug resistance in their country, the exclusions of these patients can affect the result of this paper. Even if they wish to examine only patients with bacteria sensitive to antituberculosis drugs, the numbers should be mentioned so the reader would understand the total picture. 

 From the paper, I understand there are about 1200 new patients with tuberculosis in their Oblast. This calculation is based on the population in the province and the incidents estimate the authors provide. They find only 562, and of those, 380 were expected to complete the treatment. The authors should explain the discrepancy between what was expected and what was found.

 Patients who were lost to follow-up were excluded from their analysis. This is not how it should be done according to the latest WHO recommendations. Also, the authors should do some sensitivity analysis on data. So, if we include those who were missing and those who were transferred out, the treatment complete rate is only 56%. 

 In the discussion, the authors do not discuss these major weaknesses and how they analysed the data. In addition, they need to explain the seeming discrepancy between incident cases during the year and the numbers they analysed.

I confirm that I have read this submission and believe that I have an appropriate level of expertise to state that I do not consider it to be of an acceptable scientific standard, for reasons outlined above.

# Response

**Published:** 2018-03-06

**Authors:** Omowunmi Aibana

**Affiliations:** University of Texas Health Science Center at Houston, USA

**Comment 1:** Most of the literature is old, and the authors should use more up-to-date literature. This also includes the methods and how to calculate default rates. The authors chose a method whereby they exclude patients who were lost to follow-up. However, this is a too simplistic approach. The authors should read the latest WHO recommendations on doing such calculations. 

**Response 1: We have included updated references (refs 1, 4 - 14) in our background discussion of known patient predictors of poor drug sensitive TB treatment outcomes. The data we provided in the introduction on treatment outcomes for new DSTB patients in Ukraine is the most recent WHO reported information available for the country. The WHO now reports treatment outcomes for new and relapsed cases combined but we did not evaluate TB relapses in this study for comparison.**

**Please note that treatment outcomes for DSTB in this study were categorized according to the latest WHO recommendations, which includes considering a patient to be lost to follow up (previously defaulted)** **if he or she interrupts treatment for two or more consecutive months. Therefore, we did not exclude patients lost to follow up (per WHO definitions) in our main analysis. As indicated below, we excluded patients whose final treatment outcomes were not assessed either because they transferred out and had missing outcome data. We have addressed this issue of handling missing outcome data in comment 2 below.**

**We have further replaced “default” with “lost to follow up” throughout the document to clarify that patients lost to follow up according to WHO definitions were not excluded from our analysis**

**Comment 2:** The biggest problem with this paper is how they handle missing information. They state that: "We analysed only patients with confirmed drug sensitive pulmonary TB, and we excluded from the analysis patients who transferred out or had missing outcomes."  Also, as there is a high prevalence of drug resistance in their country, the exclusions of these patients can affect the result of this paper. Even if they wish to examine only patients with bacteria sensitive to antituberculosis drugs, the numbers should be mentioned so the reader would understand the total picture. 

**Response 2: A survival analysis approach would have allowed us to evaluate the relative contributions of all patients (including those whose outcomes were not assessed) in our assessment of determinants of treatment outcomes. However, as noted in paragraph 3 of the statistical analysis section,** **the exact dates of treatment outcomes or last follow up visits were not captured in the electronic database, therefore we were unable to perform such analysis.**

**As an alternative, we have conducted a sensitivity analysis where we considered patients whose treatment outcomes were not assessed (i.e. transferred out and missing outcome data) as having a poor outcome. The results in this analysis do not differ from our main analysis; we found that patients with HIV and smear positive disease at baseline remained at increased risk of poor outcomes while high alcohol consumption conferred a modest risk of poor outcomes.**

**We have added Table 5 to depict these results and we included discussion of this sensitivity analysis in the methods and results section as follows:**

**Methods (paragraph 4 of statistical analysis): “In a sensitivity analysis, we categorized patients whose treatment outcomes were not assessed (transferred out and missing final outcome data) as having poor outcomes.”**

**Results (paragraph 6): “When we categorized patients with missing outcome data and**

**patients who transferred out as having poor outcomes, the results did not differ from the findings in our main analysis of predictors of poor treatment outcomes (Table 5).”**

**Please note that we have separately published results from a cohort of drug resistant TB patients in Kyiv Oblast [1]. Therefore, our primary aim in this manuscript was to report on predictors of poor outcomes among patients with confirmed drug sensitive pulmonary TB.**

**We have now included in the methods section (paragraph 1 of statistical analysis) the following: “** **We have previously reported on a cohort of approximately 600 patients initiated on treatment for drug resistant TB in Kyiv Oblast between 2012 and 2015”.**

**1. Aibana O, Bachmaha M, Krasiuk V, Rybak N, Flanigan TP, Petrenko V, et al. Risk factors for poor multidrug-resistant tuberculosis treatment outcomes in Kyiv Oblast, Ukraine. BMC Infect Dis. 2017;17(1): 129.**

**Comment 3:** From the paper, I understand there are about 1200 new patients with tuberculosis in their Oblast. This calculation is based on the population in the province and the incidents estimate the authors provide. They find only 562, and of those, 380 were expected to complete the treatment. The authors should explain the discrepancy between what was expected and what was found.

**Response 3: Please note that the TB notification rates we reported for Kyiv Oblast is for all pulmonary TB diagnosed and therefore includes both sensitive and resistant TB cases. We have now also reported the number of MDR-TB patients initiated on treatment in Kyiv Oblast as indicated above in response 2. Any remaining discrepancy in the expected number of TB cases might be related to lack of active screening.**

**In addition to providing MDR-TB numbers for reference, we have now included a discussion (paragraph 4) about lack of active case finding in this setting, which may contribute to under-notification of TB.**

**“We found that majority of TB patients were identified through passive case finding, which may also contribute to poor treatment outcomes. The WHO recommends systematic screening for active TB as a complement to passive case finding [1]. Studies have also shown that active TB case finding results in early detection and reduces risk of extensive disease at diagnosis [2-6], which may potentially decrease risk of poor outcomes. Active case finding also reduces risk of TB transmission [6-9] and may contribute to reducing TB prevalence.** **Lack of active case TB finding may further result in under notification of TB in this setting.** **TB control efforts in Ukraine will likely benefit from strengthening and improving health systems capacity for active case finding.”**

1. World Health Organization. Systematic screening for active tuberculosis. Principles and Recommendations. 2013. Available: http://apps.who.int/iris/bitstream/10665/84971/1/9789241548601_eng.pdf?ua=1 Accessed 20 February 2018.    

2. den Boon S, Verver S, Lombard CJ, Bateman ED, Irusen EM, Enarson DA, et al. Comparison of symptoms and treatment outcomes between actively and passively detected tuberculosis cases: the additional value of active case finding. Epidemiol Infect. 2008;136(10): 1342-1349. 

3. Eang MT, Satha P, Yadav RP, Morishita F, Nishikiori N, van-Maaren P, et al. Early detection of tuberculosis through community-based active case finding in Cambodia. BMC Public Health. 2012;12: 469.

4. Datiko DG, Yassin MA, Theobald SJ, Blok L, Suvanand S, Creswell J, Cuevas LE. Health extension workers improve tuberculosis case finding and treatment outcome in Ethiopia: a large-scale implementation study. BMJ Glob Health. 2017;2(4): e000390.

5. Kranzer K, Afnan-Holmes H, Tomlin K, Golub JE, Shapiro AE, Schaap A, Corbett EL, Lönnroth K, Glynn JR. The benefits to communities and individuals of screening for active tuberculosis disease: a systematic review. Int J Tuberc Lung Dis. 2013 Apr;17(4):432-46.

6. Cheng S, Chen W, Yang Y, Chu P, Liu X, Zhao M, et al. Effect of diagnostic and treatment delay on the risk of tuberculosis transmission in Shenzhen, China: an observational cohort study, 1993-2010. PLoS One. 2013;8(6): e67516.

7. Lin X, Chongsuvivatwong V, Lin L, Geater A, Lijuan R. Dose-response relationship between treatment delay of smear-positive tuberculosis patients and intra-household transmission: a cross-sectional study. Trans R Soc Trop Med Hyg. 2008;102(8): 797-804.

8. Fox GJ, Barry SE, Britton WJ, Marks GB. Contact investigation for tuberculosis: a systematic review and meta-analysis. Eur Respir J. 2013;41(1): 140–156.

9. Jia ZCS, Ma Y, Zhang Y, Bai L, Xu W, He X, et al. Tuberculosis burden in China: a high prevalence of pulmonary tuberculosis in household contacts with and without symptoms. BMC Infect Dis. 2014;14: 64.

**Comment 4:** Patients who were lost to follow-up were excluded from their analysis. This is not how it should be done according to the latest WHO recommendations. Also, the authors should do some sensitivity analysis on data. So, if we include those who were missing and those who were transferred out, the treatment complete rate is only 56%. 

**Response 4: Please see responses 1 and 2 above where we clarified the definition of lost to follow up and presented the suggested sensitivity analysis.**

**Comment 5:** In the discussion, the authors do not discuss these major weaknesses and how they analysed the data. In addition, they need to explain the seeming discrepancy between incident cases during the year and the numbers they analysed.

**Response 5: We have addressed the analysis of missing data and the discrepancy between expected and analyzed cases as indicated above. We have expanded the discussion on limitations to include:**

**“We also found a high proportion of patients did not have their treatment outcome assessed, which further reduces the rate of successful treatment outcomes in this setting. However, when we included patients with missing data on final outcomes in the analysis, our results did not change; we identified the same patient predictors of poor treatment outcomes.”**
